# Modern Methods in the Treatment of Skin Diseases*Read before Georgia Medical Association, Augusta, April 15, 1896.

**Published:** 1896-05

**Authors:** Bernard Wolff

**Affiliations:** Atlanta, Ga.


### MODERN METHODS IN THE TREATMENT OF SKIN DISEASES.*

BY BERNARD WOLFF, M.D.,

ATLANTA, GA.

The progress of medical science in the last score of years has been as marked in the province of dermatology as in other branches. The classification of skin diseases has been simplified. New groups have been formed as more has been ascertained of the members of them. The advance of the therapeutics of this branch has been particularly energetic and conspicuous. New remedies of great value have been introduced, approved methods of applying them have been employed, until now the dermatologist has the means at his disposal to combat with greatly increased prospect of success the various diseased conditions of the integumentary system which he encounters.

It is impossible, in a paper of such narrow limits, to more than glance at the most salient features of this progress, and to bring to your attention a brief account of the methods now in vogue in the treatment of the commoner forms of skin disease.

The remedies selected for use in cases of disease in the skin can be employed in several ways, as in baths, lotions or washes, ointments, pastes, plasters, and soaps.

Balnea, or baths, are of two kinds, the vapor and the simple medicated bath made to resemble natural mineral waters. The chief purpose of the bath is, besides cleanliness, to bring about a

*Read before Georgia Medical Association, Augusta, April 15, 1896.

healthy activity of the skin, to remove scales, soften infiltrated areas, increase the elimination of waste products through the skin, and to relieve irritable conditions of the skin. Baths are especially valuable in the treatment of parasitic and syphilitic affections.

Vapor-baths are carried out by means of bath-cabinets, in which the patient sits and is subjected to the action of the remedy in a vaporized state, at a temperature of 90 to 100° F., for fifteen minutes or longer. Various drugs may be added to the bath, the chief ones being tar, sulphur, and calomel.

The simple liquid bath has a wide range of usefulness. Starch, tar, sulphur preparations, salt, and a number of other substances may be added to the water to meet the requirements of the case. The continuous water bath of several weeks' duration has been recommended by the elder Hebra for the relief of some inveterate skin affections, as pityriasis rubra, psoriasis, etc.; but such a plan has failed to become popular in this country. Natural mineral water baths, so highly regarded by the laity, are of very questionable value as curative agents in skin diseases.

Lotions are preparations in which the remedy or remedies are held in a state of solution or suspension in water, alcohol, or oil. They are best adapted to be used in the acute and subacute states of disease of the skin. To get the full effect, the lotion should be kept in constant contact with the seat of the lesion by the application of cloths soaked in the solution and bound to the part, or by very frequently repeated sponging or moistening of the part. Lotions are of great value in the relief of itching and irritability of the skin and in squamous affections of the scalp. They are soothing, stimulating, emollient, etc., as the case may be. The remedies best adapted to use in the form of lotions are the class of antiseptics, antipruritics, reducing agents, as sulphur, ichthyol, resorcin, and the parasiticides.

Ointments and pastes are more used than any other forms of preparation in the therapy of skin diseases. The base of an ointment is a matter of importance. Upon it largely depends the benefit to be derived from the medicament. Formerly only lard and wax were used. Now we have a greater number to choose from, and more elegant vehicles. Of these vaseline, agnine, lanolin, adeps lanæ, and resorbin are the most useful. Agnine is obtained from the fat of sheep's wool. It is cheap, but has a disagreeable odor and an unattractive color. Lanolin is purified agnine. It is ivory-colored, stable, very difficult to saponify, mixes readily with water, does not become rancid, and is said to possess the quality of being absorbed through the skin to a remarkable degree. It is rarely used alone, being too tenacious, so that it must be thinned with water, oil, or vaseline. It has slight curative powers, and is bland and unirritating. Adeps lanæ has properties similar to lanolin, but differs from it in being a definite chemical product.

Ledermann (*Monatshefte für Prakt. Derm.*, July 15, 1894,) introduced a new excipient, called by him resorbin. It is composed of refined almond oil emulsified with distilled water, with the addition of a small quantity of yellow wax and lanolin. It is claimed to possess to a very high degree the power of penetrating the skin, very slight friction being required.

Casein ointment has been put upon the market by Beiersdorf, of Hamburg, on the suggestion of Unna. It is very elegant in appearance, but my slight experience with it has led me to regard it as being incompatible with too many substances to be of much practical value.

Ointments, with whatever base, may be cooling and lubricant, penetrating, stimulating, or continuous, as Hebra's diachylon ointment, which is scarcely more than a spread plaster.

To overcome the objections of uncleanliness and lack of sufficient adhesive power of ointments to secure constant and intimate contact with the afflicted areas, various pastes of puffy-like consistence have been devised. Pastes resemble ointments in the facility with which they may be applied, but differ from them in becoming dry and adhering firmly. One of the most generally useful is Lassar's paste, the formula for which is:

℞ Acid salicylic.................................................gr. x.

Vaseline.................................................℥ ss.

Zinc oxid,

Pulv. amyli āā.................................................ʒ ij. *M.*

Boracic acid may replace the salicylic or resorcin, as in Ihle's modification. This paste is of great utility and can be used on any part of the body with the exception of the palms, soles, and eyelids. The basis of this paste is starch and vaseline. Instead of starch may be substituted infusorial or diatomic earth, which is extremely porous and makes a fine homogeneous paste with vaseline.

The following paste is of great value in the treatment of seborrheal affections:

| | | | |
|---|---|---|---|
| ℞ | Zinc oxid | parts 6 | |
| | Sulph præcip | " 4 | |
| | Terræ siliceæ | " 2 | |
| | Adipis benzoat | " 28 | *M.* |

However valuable these pastes may be, they cannot approach in usefulness the gelatine preparations of Pick and Unna.

Unna's formula for zinc jelly is the following:

| | | | |
|---|---|---|---|
| ℞ | Gelatin | 15.0 | |
| | Zinc oxid | 10.0 | |
| | Glycerin | 30.0 | |
| | Aquæ | 40.0 | *M.* |

When cautiously melted and combined this makes a mass of leathery consistence—very pliant. When about to be used, it must be placed in a vessel and immersed in hot water until it is liquefied. It is then painted on the surface with a stiff brush, and dabbed with a bit of loose cotton to facilitate hardening. When hard, it is smooth, dry, pliant. It can be used in any locality, and is extremely efficient. It excludes air, and hastens the absorption of infiltrated areas, allays irritation, and promotes healing. It is to be most highly recommended in the treatment of ulcers of the leg and all stages of eczema in all localities. The cardinal principle of the therapeutics of skin disease is to secure absolute and continuous application of the remedy to the affected parts, otherwise the treatment will be very slow, or altogether ineffectual. Pastes come very near fulfilling this requirement; but there are still better means of accomplishing this aim, and doing it in a much more thorough manner. Passing over the use of collodion as a vehicle, and traumaticine, a solution of gutta-percha in chloroform, we come to what is, without doubt, the ideal method of applying a remedy in the treatment of skin diseases. It is found in the gutta-percha mull, which belongs to the class of, but is much superior to, the ordinary spread plasters. In the gutta-percha plaster mull the medicament is dissolved in a minimum quantity of a suitable vehicle, and is spread on an extremely thin layer of gutta-percha, backed by coarse gauze. The medicament is so disposed throughout the mass that the smallest square of a meter roll will contain its exact proportion of the total quantity of medicament in the meter roll. An exact dosage is thus secured. They are easily applied. The protective gauze is removed and the surface of the plaster is applied directly to the skin, holding it there for a short time until the body-heat affixes it. I have not time sufficient to devote to a detailed account of the manifold good effects of this ideal preparation. Suffice it to say that it may be used anywhere on the body, and with the appropriate remedy incorporated, in any disease of the skin which is not too extensive. It fulfills all indications for treatment in the way of insuring close and prolonged contact, deep and powerful therapeutic effect, and is moreover cleanly in the extreme. The sole obstacle to its general use is its expense. If carefully and economically used, this will not be regarded as an insuperable obstacle.

The impetus given to the study of animal extracts by Brown-Séquard, Pasteur, Hammond, and others has been felt in the province of dermatology. Thyroid extract, which has effected such miraculous cures in the treatment of myxedema and cretinism, has been given a thorough test of its value as a curative agent in certain intractable skin diseases.

The use of soaps is of some importance in the treatment of diseases of the skin. Only neutral soaps, or those containing an excess of unsaponified fat, are to be recommended. Free alkali is destructive to the protective, horny layer of the skin. The over-fatty soaps make commendable vehicles for various medicaments, the most frequently used of which are hydronaphthol, resorcin, sulphur, tar, corrosive sublimate. They act as emollients, and are of value only as adjuncts to more energetic treatment. The use of soaps has the effect to encourage cleanliness on the part of the patient and cleanliness is one of the prerequisites of successful treatment. The results, with an exception in the case of lupus, which seems to have been somewhat influenced, have been entirely negative. Psoriasis and xeroderma have not been benefited by its use, contrary to expectation. Tuberculin has been tried in tuberculosis of the skin, but has now been practically abandoned.

Electricity as a therapeutic agent has a place in resources of the dermatologist. Good effects have been observed from the use of the current from the static machine in cases of eczema, and galvanism has been successfully employed in the treatment of acne, alopecia areata and a number of other cutaneous disorders. The principal use for electricity in the province of skin diseases is found in its employment for the removal of superfluous hairs and of new growths of small extent. Electrolysis offers the only certain means of completely and almost painlessly destroying hairs and preventing their recurrence. A little experience with the use will readily demonstrate the fact. I use instead of the ordinary steel needle the bulbous-pointed needle devised by the late P. S. Hayes, known under his name. The blunt point removes, to a large extent, the danger of piercing the wall of the hair follicle, an accident that is very apt to occur in using the sharp needle, in which case the papilla is not reached and the treatment fails of its purpose. The removal of nævi, moles, warts, and small malignant new growths of the skin by electrolysis is a well established surgical procedure.

The above brief résumé of the modern methods of treatment of skin disease will, I trust, indicate in some measure the progress which has been made. There is need of much more before we shall be contented with the methods at our command. It is to be hoped that continuous research along this line will be fruitful of results in the future as it has been in the last decade.

---

"I AM not always in a great hurry to operate in appendicitis, but I am more inclined to wait for the more acute symptoms to wear off, and operate, if at all, after suppuration has taken place, or during the quiescent stage between the attacks. I wish my voice was strong enough, just here, to call a halt to the men who say, 'Operate at once—not this afternoon or to-morrow, but now,' in all cases when the disease is recognized."—*McGuire.*

